# Chemokines

**DOI:** 10.1100/tsw.2007.6

**Published:** 2007-02-19

**Authors:** Richard Horuk

**Affiliations:** Department of Immunology, Berlex Biosciences, Richmond, CA, USA

**Keywords:** Chemokine, autoimmunity, GPCR, multiple myeloma, endometriosis, antagonist

## Abstract

Chemokines are a family of polypeptides that direct the migration of leukocytestoward a site of infection. They play a major role in autoimmune disease and chemokine receptors have recently been found to mediate HIV-1 fusion. In this short review we examine the role of chemokines in host defence and in the pathophysiology of autoimmune diseases. We conclude by discussing various therapeutic approaches that target chemokine receptors and that could be beneficial in disease.

## INTRODUCTION

The human body is constantly being exposed to an ever-changing milieu of microorganisms. Many of these, such as the commensal gut bacteria that peacefully coexist with us, are not harmful and can even be beneficial. However, others that include a large number of pathogenic protozoa, bacteria, and viruses are harmful and would destroy us if left unchecked. To deal with this dangerous army of invaders, we have evolved a very complex defense system known collectively as the immune system. A critical component of the host defense system is a family of proteins known as the chemokines (figure 1)[1]. These proteins are small, mainly basic molecules that bind to G-protein coupled receptors (GPCRs) and initiate the chemotaxis and directed migration of immune cells from the blood and lymph into the tissues where they can be mobilized to seek out and destroy the foreign invaders[1]. This search and destroy method is extremely efficient and has evolved over many thousands of years to deal with an ever-changing burden of disease-bearing organisms. However, it does have its dark side in that the very cells that protect us can, for a variety of reasons, sometimes turn on us and destroy our own cells and organs by friendly fire, giving rise to the concept of autoimmunity. For example, immune cells can be triggered to destroy the protective myelin sheath that surrounds neurons so that eventually the neurons die. The consequence of this manifests itself as multiple sclerosis. Infiltrating T lymphocytes can seek out and destroy pancreatic beta cells, destroying them and reducing the ability to produce insulin. The consequence of this will eventually give rise to type I diabetes. Thus, chemokines are like the Roman god Janus who presents two distinct faces, one beneficial and one destructive. It is this destructive face of the chemokines that initially attracted the attention of the pharmaceutical industry since the chemokines mediate their function by binding to and activating GPCRs, which are by themselves one of the most exploited families of therapeutically useful proteins.

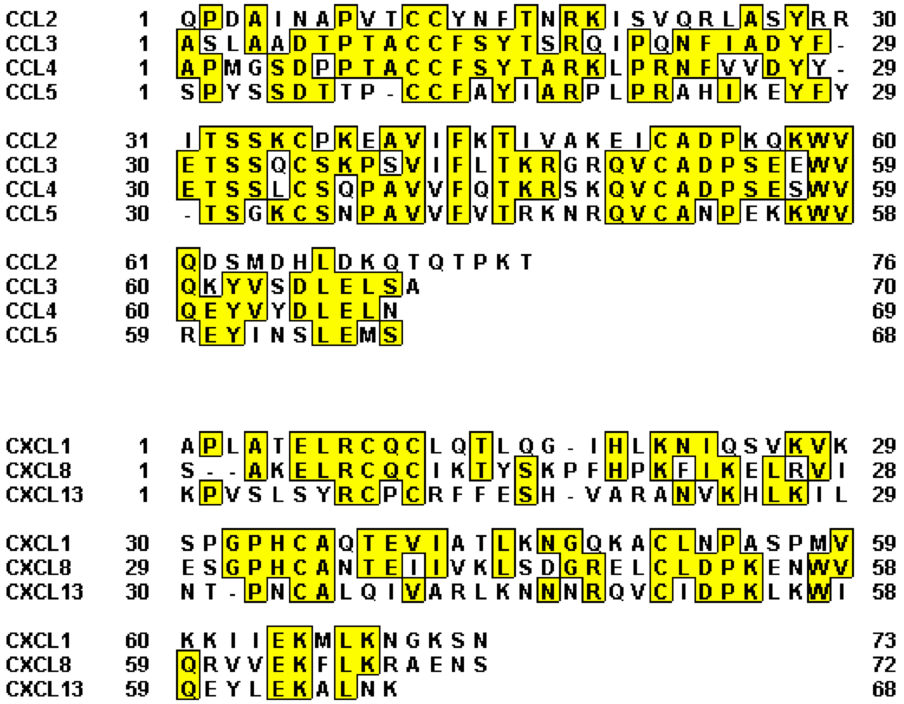

**FIGURE 1.** Primary structure of selected members of the CC and CXC chemokine superfamily. Chemokines have been grouped into the CXC, CX3C, CC, and C branches of the family based on the position of the conserved four cysteine motif. Conserved amino acids are shown in yellow.

So far eighteen different chemokine receptors have been cloned (Figure 2) and well over 40 different chemokines belonging to four separate classes, CC, CXC, CX3C and C, depending on the nature and disposition of the invariant cysteines in their sequence(2).

Most of the chemokine receptors were identified and cloned from immune cells, which are their major target cells. In addition, several virus-encoded proteins that have sequence homology and share the serpentine structure of the cloned chemokine receptors have been identified. These viral proteins have been termed viroceptors and appear, in some cases, to function as chemokine receptors[3]. Finally, a protein first identified in human erythrocytes as a CXCL8 binding protein has been shown to be a novel chemokine-binding protein that binds both CC and CXC chemokines with high affinity[4]. This protein, known as DARC, is identical to the Duffy blood group antigen a receptor for the malarial parasite *Plasmodium vivax* .

Chemokine receptors belong to the rhodopsin family of GPCRs that signal through coupled heterotrimeric G proteins. At the latest count over 450 members of this superfamily have been identified and classified into families(5). Six CXC, ten CC, one C and one CX3C chemokine receptors have been cloned so far.

Although each of these receptors binds only a single class of chemokines, they can bind several members of the same class with high affinity. Only the promiscuous chemokine-binding protein DARC[4] and the viral chemokine-binding protein M3[6] have been shown to bind both CC and CXC chemokines with equal affinity.

Although leukocytes continue to be the major site of expression of chemokine receptors, several studies have recently demonstrated chemokine receptor expression on neurons in the CNS[7]. A number of chemokine receptors including CXCR2, CXCR4, CCR1, CCR5, and DARC have been demonstrated in either adult or in fetal brain. Not only were these receptors present on the cell surface, but they were also functional. Clearly, the role of these receptors on CNS neurons must be very different from their role

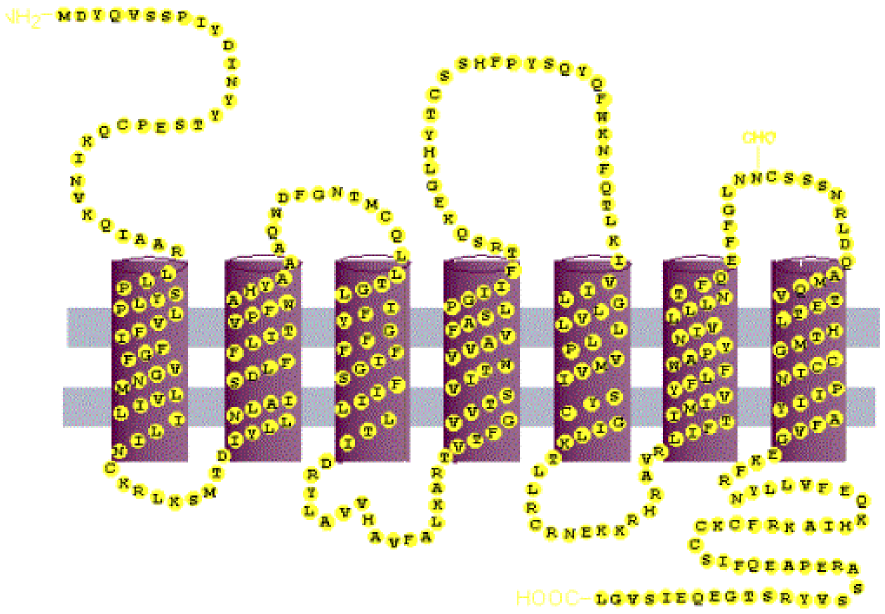

**FIGURE 2.** Proposed membrane topography of the human CCR5 receptor. Membrane spanning alpha helices are defined based on hydropathy analysis on immune cells. Given that human astrocytes can be stimulated with cytokines to upregulate the expression of chemokines, it is tempting to speculate that during the development of the CNS that chemokines secreted by astrocytes can engage specific receptors expressed on neurons and may play a role in the directed migration of specific subsets of neurons to distinctive regions of the brain.

Genetic mutations of receptors, both natural and induced (by targeted gene disruption), can help to unravel their biological roles. Nature has been generous in this regard by providing us with two naturally occurring examples of gene inactivation for chemokine receptors. Humans homozygous for inherited inactivating mutations of the Duffy (DARC) gene[8] and the CCR5 gene[9] have been identified, and appear to be phenotypically normal and healthy. Indeed, these gene inactivations appear to be beneficial to their hosts, rendering them resistant to certain infectious diseases. For example, the Duffy-negative individuals are resistant to malaria induced by *P. vivax*, which utilizes DARC to attach to and enter erythrocytes[10], and the CCR5 negative individuals are resistant to HIV-1, which utilizes this chemokine receptor as a coreceptor for invasion[9].

Analysis of receptor-inactivated individuals can also be useful to clarify their role in disease. For example, a variety of studies[11,12,13,14,15] suggest that expression of the CCR5Δ32 mutation, which results in a truncated allele of the CC chemokine receptor CCR5 gene encoding a nonfunctional receptor, does not confer protection from multiple sclerosis. In some studies, however, CCR5Δ32 was associated with a lower risk of recurrent clinical disease activity[12]. Specifically, these studies seemed to indicate that the age of onset of disease was approximately 3 years later in patients carrying the CCR5Δ32 deletion ($p$ = 0.018 after controlling for gender effects). Thus, chemokine receptor expression may be associated with differential disease onset in a subset of patients and may provide a therapeutic target to modulate inflammatory demyelination. These results directly link intrathecal inflammation to disease activity in patients with multiple sclerosis, suggesting that treatments targeting CCR5 may attenuate disease activity in multiple sclerosis[16].

Somatic mutations in GPCRs have also yielded valuable insights into receptor regulation and function. For example, polymorphisms in the amino acid sequence of some GPCRs have led to constitutively active receptors. A recently described chemokine receptor encoded by a Kaposi's sarcoma–

associated herpesvirus, HHV8, falls into this class[3]. This virus has been shown to encode a constitutively active (agonist-independent) chemokine receptor that is a potential oncogene. Receptor signaling leads to cell transformation and tumorigenicity, and induces a switch to an angiogenic phenotype. This receptor can activate protein kinases by triggering signaling cascades similar to those induced by inflammatory cytokines (vascular endothelial cell growth factor) that are known activators of angiogenesis. Elucidation of the mechanism by which this receptor constitutively signals will undoubtedly aid in an understanding of chemokine receptor function.

## CHEMOKINES IN DISEASE

As already briefly discussed above, chemokines play a role in a variety of autoimmune and inflammatory diseases including multiple sclerosis, rheumatoid arthritis, diabetes, multiple myeloma, endometriosis, and organ transplant rejection. Rather than discussing each of these in turn, we will illustrate their potential in disease by reference to multiple myeloma and endometriosis.

### Multiple Myeloma

Multiple myeloma is a disease characterized by the clonal expansion of plasma cells in the bone marrow and is responsible for about 1% of all cancer-related deaths in Western countries[17]. A major clinical feature of multiple myeloma is the development of osteolytic bone disease characterized by the presence of bone pain, hypercalcemia, and pathological fractures[17]. Bone destruction is a common manifestation of the disease and is a major source of morbidity for these patients. Bone destruction results from increased osteoclastic bone resorption and decreased bone formation that occur only in areas of bone adjacent to myeloma cells[18]. These data suggest that the bone disease results from local production of an osteoclast stimulatory factor (OSF) that is secreted by myeloma cells, marrow stromal cells, or both. Although the identity of this factor(s) *in vivo* is currently unknown, one molecule that has been implicated in the development of this bone disease is the chemokine CCL3[19,20], which is a ligand for the CC chemokine receptors CCR1 and CCR5.

In the first of two separate studies, Choi et al.[19] identified CCL3 as the OSF present in patients with multiple myeloma. They showed that CCL3 is an OSF in human marrow cultures and that it is overexpressed in patients with multiple myeloma, but not in controls. In addition, a neutralizing antibody to CCL3 blocked the OSF activity present in bone marrow plasma from multiple myeloma patients. These data suggest CCL3 may be a major mediator of the bone destruction seen in patients with multiple myeloma.

In a second study, Choi et al.[20] investigated the role of CCL3 in multiple myeloma bone disease *in vivo*. A human multiple myeloma–derived cell line stably transfected with an antisense construct to CCL3 was tested for its capacity to induce multiple myeloma bone disease in SCID mice. Human CCL3 levels in marrow plasma from these mice were markedly decreased compared with controls treated with a cell line transfected with an empty vector. Mice treated with CCL3 antisense cells lived longer than controls and, unlike the controls, they showed no radiologically identifiable lytic lesions. Furthermore, antisense to CCL3 blocked the adherence of myeloma cells. CCL3 increases $\beta_1$ integrin expression on multiple myeloma cells and increased adherence of multiple myeloma cells to marrow stromal cells. These data strongly suggest an important role for CCL3 in cell homing, survival, and bone destruction in multiple myeloma.

In summary, these studies have demonstrated that CCL3 is an osteoclast-stimulating factor *in vivo* and that antisense to CCL3 decreases tumor burden and bone destruction in a mouse model of multiple myeloma. In addition, blocking CCL3 *in vivo* decreases bone destruction and myeloma tumor burden by decreasing multiple myeloma cell adherence to marrow stromal cells. From recent studies examining the expression of chemokine receptors in multiple myeloma cells[21], it appears that CCR1 is expressed in these cells. Furthermore, treatment of these cells with CCL3 and CCL5 induced calcium transients and cellular migration, indicating that the cells expressed functional CCR1. Thus, since CCR1 appears to be the major chemokine receptor that is involved in mediating these effects, it seems reasonable to conclude that blocking it should be useful therapeutically to treat multiple myeloma.

## Endometriosis

Endometriosis is a chronic inflammatory disease, characterized by implantation and growth of endometrial tissue outside the uterine cavity[22]. It is classically described as the presence of endometrial tissue (glandular epithelium and stroma) outside the uterine cavity. Endometriosis is a benign chronic inflammatory disease that affects 15–20% of all women in their reproductive life. Retrograde menstruation is postulated as the initiating event in the pathogenesis of the disease, and this is accompanied by an intraperitoneal infiltration of the lesions by macrophages and T cells. The recruitment of these leukocytes into the endometrial lesions is initiated by the local production of chemokines. The most notable example is the CC chemokine CCL5[22], which is, among others, a ligand for the chemokine receptors CCR1 and CCR5. A recent study using real-time PCR and FRET technologies to genotype and evaluate the CCR5Δ32 failed to demonstrate any association of this polymorphism in the pathophysiology of endometriosis in the population that was examined[23]. As the authors indicate, although these data do not completely rule out a possible role of other genetic CCR5 variants in this pathology, it remains less likely. Based on data from a variety of studies that suggest a role for CCL5 in the pathophysiology of the disease[22,24,25,26], it is tempting to speculate that chemokine receptor antagonists that target CCR1 might be beneficial in treating this disease.

## CHEMOKINE RECEPTOR ANTAGONISTS

Type the search term “chemokine receptor antagonists” into PubMed and you will retrieve well over 1300 citations. Similarly, the same search in U.S. patent applications yields well over 500 hits for issued patents and over 1300 citations for patent applications. This proliferation of interest in the chemokine receptors as drug targets was fueled by the discovery in 1995 that the chemokine receptors CCR5 and CXCR4 were able to act as coreceptors that were required for cellular entry by HIV-1[27,28]. CCR5 is an entry cofactor for M-tropic isolates of HIV-1 and is important in the early proliferative part of the disease, while CXCR4 is a coreceptor for T-tropic isolates of HIV-1 whose emergence in infected individuals usually correlates with accelerated disease progression. It is convenient at this point to illustrate the progress that chemokine receptor antagonists have made with reference to the HIV coreceptor CCR5 since this is the most advanced chemokine receptor antagonist program.

HIV-1 entry into a cell comprises at least three separate events[29]: (1) the virus attaches itself to the host cell by means of an initial high-affinity interaction between viral envelope protein and CD4, (2) this is followed by a subsequent interaction with an appropriate chemokine receptor that triggers the final conformational changes in envelope protein, and (3) this culminates in fusion between the now exposed viral fusion protein and the host cellular membranes, which then allows infection to proceed. It is the second step in this process that CCR5 and CXCR4 inhibitors interfere with. A number of pharmaceutical companies including, but not limited to, Pfizer, Schering Plough, GSK, Millennium, Merck, Takeda, Novartis, and Astra-Zeneca have programs aimed at identifying CCR5 antagonists. Of these, the Pfizer, Schering Plough, and GSK programs are the most advanced in various stages of clinical development and are described below.

Although there is limited information available on many of these programs, Pfizer appears to have the most advanced program and their CCR5 antagonist UK-427857 (maraviroc) entered phase II/III trials in April 2005 for the potential treatment of HIV infection in over 1300 HIV-positive patients infected with CCR5-tropic virus strains[30,31]. This small molecule (Fig. 3, compound 1) blocks viral replication and prevents gp120 binding with CCR5 ($IC_{50}$ 0.2 and 43 n*M*, respectively) *in vitro*. Clinical data for

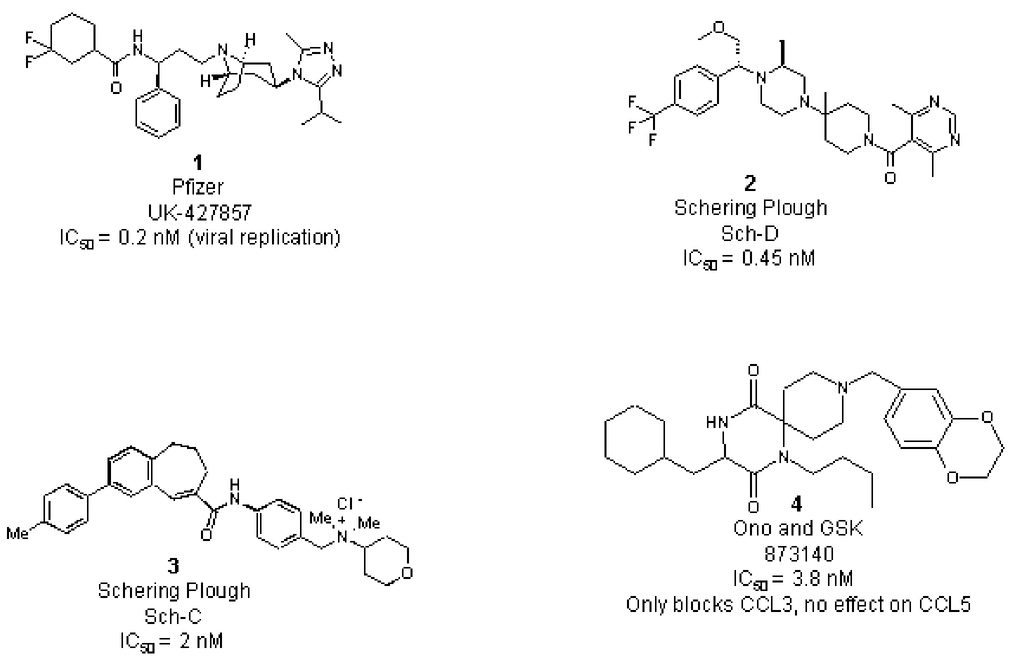

**FIGURE 3.** Structures of CCR5 antagonists - $IC_{50}$ reported for inhibition of $^{125}I$ -CCL5 binding to CCR5 expressing cells.

UK-427857 shows that doses of 300 mg twice daily were well tolerated and no serious adverse events were reported. UK-427857 given as a monotherapy for 10 days reduced HIV-1 viral load by up to 1.6 log orders, without significant side effects. However, one patient receiving a cocktail of maraviroc, 300 mg once daily (blinded study drug), and zidovudine/lamivudine and other toxic drugs had to receive a liver transplant after five maraviroc doses, but it is likely that this is not due to the CCR5 inhibitor; rather, it could be attributed to the toxic nature of the drug regimen the patient was receiving.

Schering Plough entered phase I clinical trials with their antagonist (Fig. 3, compound 2) in 2001 and an update on the development of their small molecule CCR5 antagonist, Sch-D (vicriviroc), was recently given at a clinical meeting[32]. This small molecule has completed phase I for the potential treatment of HIV infection. Sch-D is the lead compound in a series of second-generation CCR5 antagonists described by Schering Plough. This compound was well tolerated in a study with 48 patients that were chronically infected with HIV. In phase I, patients were administered Sch-D at rising doses of 10, 25, and 50 mg twice a day for 14 days. A dose-dependent reduction in viral load was observed and the average fall in HIV particles was at least 1 log in each treatment group. The compound is significantly more potent *in vitro* (up to tenfold greater activity, $IC_{50}$ 0.45 n*M*) than Sch-C (Fig. 3, compound 3) and had no cardiovascular side effects[33]. The compound had a good pharmacokinetic profile, 100% bioavailability and 84% protein binding with good CNS penetration, and did not cause inhibition or induction of liver enzymes[33]. Based on these positive data, vicriviroc entered phase II clinical trials in April 2005 in over 90 HIV-positive patients infected with CCR5-tropic virus strains[34]. The patients were given cocktails of either efavirenz or vicriviroc together with AZT/3TC. Unfortunately, the clinical trial was halted after 24 weeks due to a lack of efficacy with the vicrivoc group. The lack of efficacy in the vicrivoc group was initially attributed to drug resistance since the HIV levels in the patients' serum were observed to be increasing after about 16 weeks of treatment. However, this does not appear to be the case according to a presentation by Wayne Greaves in an oral session at the 13th CROI in Denver Feb 8, 2006. It appears from the presentation that the primary reason for the failure of the drug could be related to the fact that the drug dose used was too low, leading to a very low viral load reduction. Higher dosing of the drug (of 100 to 125 mg) can be explored, perhaps with ritonavir boosting. Apparently the trial included a 2-week monotherapy period at the start of the study that was stopped and this may have caused drug resistance to emerge, but a higher and more potent dosing regimen could address this concern. Although valuable time was lost with this poorly designed study, restudy of the drug at improved dosing appears feasible, particularly because there were no safety issues from patients treated with the drug

Ono Pharmaceuticals recently disclosed a novel spirodiketopiperazine derivative, GW873140 (Fig. 3, compound 4), that they are developing in conjunction with GSK, GSK-873140 (aplaviroc)[35]. The antagonist effectively blocked HIV-1 gp120/CCR5 binding and had potent activity against a wide range of R5 HIV-1 isolates. Aplaviroc demonstrated *in vitro* antiviral activity with an $IC_{50}$ against CCR5-tropic HIV-1 of 1 n*M*[35]. Pharmacokinetic studies revealed favorable oral bioavailability in rodents[35]. These data paved the way for the development of aplaviroc as a potential therapeutic for HIV-1 infection. Human phase I studies to investigate the safety, tolerability, and pharmacokinetics of escalating single (50–1200 mg) and repeat (200–800 mg BID) doses of aplaviroc have been conducted in 70 healthy volunteers[36,37]. The trials indicated that aplaviroc is well tolerated up to a dose of 1200 mg following single dose and 800 mg following multiple dosing twice a day[36,37]. No serious adverse events were reported, although some incidents of QTc prolongation were noted following administration of the drug in healthy individuals[36,37]. Based on these data, the drug went into phase II clinical trials in 2005, but unfortunately on September 15$^{th}$, GSK reported that these trials had been halted because of safety concerns. Numerous patients presented symptoms of severe hepatotoxicity with elevated liver enzymes (AST, ALT) and total bilirubin. In addition, GSK is amending its ongoing phase III studies in treatment-experienced patients that are still ongoing, but liver toxicity will be closely monitored. Given that no problems of severe liver toxicity were observed with the other CCR5 inhibitors from Pfizer or Schering-Plough, it is likely that the toxicity is drug related and not CCR5 related.

## CONCLUSIONS

As this review has attempted to point out, chemokines play an important role in both host defense and in autoimmunity. Because of this, they will continue to be the focus of intense research by the pharmaceutical industry. It remains to be seen whether the chemokine receptor antagonists will live up to their expectations to become fully approved drugs. If this promise is to be realized in the next couple of years or so, it will most likely be as drugs to combat HIV. The role of these chemotactic molecules in inflammation and in autoimmunity is continuously expanding and we can expect to see new approaches that might prove to be of potential therapeutic benefit in the near future.
